# Prevalence of the relative age effect among high-performance, university student-athletes, versus an age-matched student cohort

**DOI:** 10.17159/2078-516X/2022/v34i1a13310

**Published:** 2022-01-01

**Authors:** S Dube, H Grobbelaar

**Affiliations:** Division of Sport Science, Department of Exercise, Sport and Lifestyle Medicine, Faculty of Medicine and Health Sciences, Stellenbosch University, Stellenbosch, South Africa

**Keywords:** university sport, birth quartiles, selection bias, birth dates

## Abstract

**Background:**

Relative age effect (RAE) refers to the over-representation of athletes born earlier in the calendar year covering a specific sport. The RAE is especially prevalent in youth sports but often persists into senior competitive levels.

**Objectives:**

To determine the prevalence and magnitude of the RAE among student-athletes in a high performance (HP) programme at a South African university, according to year, sports code and sex, compared to the general student cohort.

**Methods:**

Cross-sectional descriptive analysis of HP-student-athletes and an age-matched student cohort from 2016 to 2021. Birthdate data were extracted for the HP student-athletes (N = 950: men = 644, women = 306) and student comparison group (N = 47 068; men = 20 464; women = 26 591; not disclosed = 13). Differences were determined using Chi-squared and Fisher’s exact test. Residuals examined relative age quartile differences. The steps were applied across academic years, sport code and sex

**Results:**

The RAE was more pronounced among the student-athletes compared to the age-matched student cohort. The RAE was occasionally observed among the HP-student-athletes; however, the prevalence was inconsistent across the respective years under investigation and only noted in certain sport codes (i.e. swimming, rugby union and cricket). There were no sex differences among the HP student-athletes.

**Conclusion:**

Where the RAE was noted, the selection bias favoured the relatively older student-athletes. The mechanisms for RAE are multifactorial and complex. A combination of factors, such as competition depth, the popularity and physicality of a sport and socialisation may be involved.

The relative age effect (RAE) refers to the over-representation of athletes born earlier in the calendar year covering a specific sport. RAE is determined by birth date concerning age-group cut-off dates.^[1]^ These differences are typically associated with short- and/or long-term effects universally known as the relative age effects (RAEs).^[2]^ The RAE is expressed by the difference between expected and the observed birthdate distributions of participants.^[3]^ Research is consistent in reporting the immediate and long-term selection, attainment and participation advantages enjoyed by relatively older participants (i.e. those born earlier in the selection year).^[3]^ These outcomes extend across developmental periods but appear to be deep-rooted and most pronounced in competitive adolescent male team sports.^[1]^

Importantly, RAEs are not reinforced by a single factor. Supported by a range of descriptive data over the last three decades, a combination of physical, psychological, motivational and socialization factors work together to produce the effect.^[1,4]^ Once participants are selected, they will have access to better coaches, training facilities, and competitive opportunities.^[5]^ This becomes a key aspect of their future sporting career, resulting in the continued prevalence of the RAE at senior sporting levels.^[5,6]^

While age-related factors are critical antecedents of RAEs, they are also reinforced by more global factors (i.e. depth of competition and the skill level required), which influence the developmental context.^[4]^ Recent evidence points to a RAE reversal at senior elite levels, suggesting that relatively younger athletes may be more likely to experience success and enjoy longer careers compared to relatively older players.^[7,8]^ One possible explanation for this reversal is that at the senior sports level and in certain individual sports, technical, tactical and psychological traits become more valued than body size.^[7]^ Once the physical advantage that relatively older individuals typically enjoy during adolescent sports is no longer prevalent, superior skills gained by relatively younger players, who persist in an unfavourable system, place them at an advantage.^[7–9]^ Being relatively younger is therefore not an automatic disadvantage for all youth sports participants. However, it is a disadvantage for most relatively younger athletes. Any reversal affects a small proportion of Q4-born athletes, whereas the overall RAE affects a much higher proportion of youth sports participants.

Whilst most RAE studies have focused on youth and professional sport,^[2]^ few studies have investigated this prevalence among university student-athletes. South Africa’s sports system is uniquely organised into competitive school and university sport which often forms part of the pathway to elite sport, compared to elsewhere in the world where club sport tends to dominate.^[10]^

Stellenbosch University has a High Performance Sports Unit that selects a limited number of student-athletes into its talent development programme each year. This stream offers differentiated experiences, including better coaches, sport science services and opportunities for televised competitions (e.g. Varsity Sport/Varsity Cup). Selection into the HP programme is prestigious and represents a facet of cultural identity, which probably proliferates competition and selection pressure.^[4]^ These factors make it a suitable environment for the RAE to be prevalent, as many participants compete for the limited number of positions and resources.^[4]^

This study aimed to determine if the RAE is prevalent among HP-student-athletes across academic years, sport codes and sex, compared to an age-matched student cohort. This is a pertinent inquiry as it highlights the magnitude of the RAE and adds to the few studies on South African student-athletes.^[11]^ The study attempts to fill this gap in the literature by focusing on the timeline and impact the RAE makes when prevalent in youth sports, and may provide insight into the selection and participation patterns of university student-athletes. The researchers hypothesised that the RAE will be prevalent and that there would be a bias towards relatively older student-athletes being selected for high-performance opportunities.

## Methods

Ethical approval was received from the SU Research Ethics Committee for Social, Behavioural and Educational Research (REC: SBE project number: 21919), and institutional permission was granted by the Division for Information Governance (IG-2166). Since the data did not contain identifiable information, informed consent was not required. The study was conducted according to the Declaration of Helsinki.

We included date of birth data of South African SU students aged 18 to 25 years from 2016 to 2021. The study was delimited to the last six years for which complete data sets exist for the student-athletes. This is when Maties Sport started the induction, monitoring, and tracking of their HP-student-athletes. Since the HP programmes focus on Varsity Sport/Varsity Cup sporting codes, 25 years was set as the maximum age for the student-athletes, coinciding with the competition age limit. Non-South-Africans were excluded to ensure that all participants were subject to the same cutoff date (1 January) used for age-group categorisation.

All 128 230 data records were analysed in RStudio. To ensure that the participants in each academic year were unique and included once only, the analysis was restricted to the new intake students for each year (N = 48 018). The data was divided into two groups: (1) general student cohort (N = 47 068; men = 20 464; women = 26 591; not disclosed = 13), and (2) HP-student-athletes (N = 950; men = 644; women = 306). The student-athletes consisted of 11 HP sport codes: Athletics = 90; Basketball = 67; Cricket = 77; Cycling = 33; Field hockey = 133; Netball = 67; Rugby union = 260; Soccer = 95; Swimming = 72; Tennis = 40; Water polo = 16). It was essential to impose a comparison cluster of aged-matched general students to assess whether the RAE was prevalent in the general student population or whether the phenomenon is sport-specific.

Starting with January, all participants were grouped into quartiles (Q1: January to March, Q2: April to June, Q3: July to September, Q4: October to December). The Chi-Square goodness of fit test was used to test differences in birth quartile frequencies of the full student population against a theoretical expected distribution, a day-corrected quartile distribution (Q1 = 24.7%, Q2 = 24.9%, Q3 = 25.2%, Q4 = 25.2%).^[3]^ Compared to a uniform distribution (25% per quartile), the day-corrected distribution accounts for the varying number of days per month.^[3]^ A series of Chi-squared tests of independence (χ2) was used to test differences in birth quartile frequencies of the HP-student-athletes against the general student cohort according to year and sex. Fisher’s exact tests were used to assess significant differences in birth quartile frequencies according to sport code.

For all analyses, a p-value of <0.05 was the criterion for a significant difference in distributions. Furthermore, Cramer’s V identified the magnitude of the effect size. A *post hoc* test to calculate the standardised residuals (SR) was used to determine which birth quartiles differed significantly from the expected distribution. Since all the quartile distribution comparisons had *df* = 3, Cramer’s V was interpreted as follows: <0.06 = trivial effect; 0.06 < V ≤ 0.16 = small effect; 0.17 ≤ V < 0.29 = medium effect; and V > 0.29 = large effect and residuals > 1.96 = over-representation, while < −1.96 = under-representation of births.

## Results

For the general student population (South African students, age-matched to the student-athlete’s Q1 = 27%, Q2 = 25%, Q3 = 25%, Q4 = 23%, a RAE was evident (χ2 = 272.42, p < 0.01, Cramer’s V = 0.02) when compared to the day count distribution. A follow up χ2 test of independence confirmed a significant association between sex and birth quarter among the student population (χ2 = 21.28, p < 0.01, Cramer’s V = 0.02) with a quartile distribution [residual] for men (Q1 = 28% [2.40], Q2 = 26% [0.75], Q3 = 24% [−1.64], Q4 = 22% [−1.70]) and women (Q1 = 27% [−2.13], Q2 = 25% [−0.66], Q3= 25% [1.46], Q4 = 23% [1.51]).

**Fig. 1:**
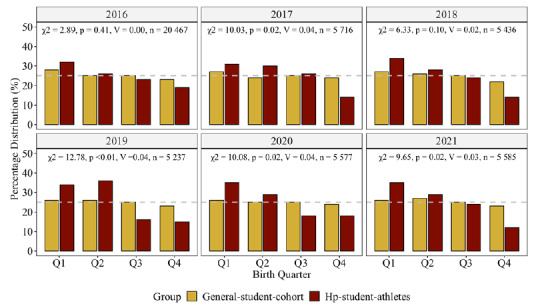
Results from the Chi-square test of independence between-groups according to the academic year. n indicates total number of students. p, χ^2^ and V values indicates birth quarter difference for the year. Dotted line at 25% indicates reference for uniform distribution.

**Fig. 2:**
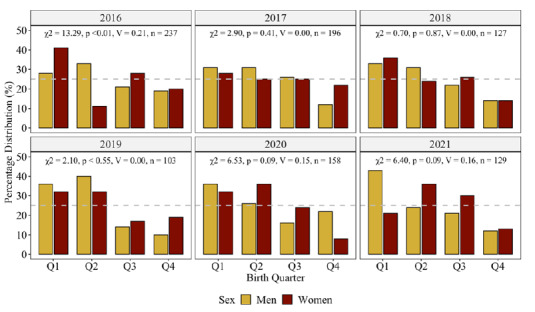
Results from the Chi-square test of independence between-sex for HP-student-athletes. n indicates total number of HP-student-athletes. p, χ^2^ and V values indicates birth quarter difference for the year. Dotted line at 25% indicates reference for uniform distribution.

**Fig. 3:**
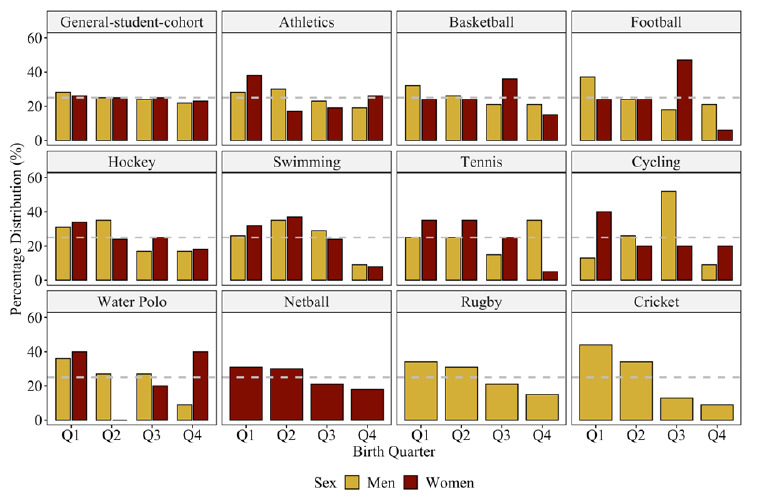
Distribution of birth quarter for each sport code by sex. Dotted line at 25% indicates reference for uniform distribution.

Table 1 contains the between-group comparison results per sex (i.e., HP-student-athlete men/women versus general-student-cohort men/women) and year. Table 2 reports the Fisher’s exact test results and residuals for each sport code. Figure 1 depicts the group differences in birthdate distribution for the HP-student-athletes and the general student cohort for each year. Figure 2 graphically illustrates the birth quartile between-sex differences among the HP-student-athletes. Figure 3 revealed the between-sex comparisons for the general-student-cohort as well as the eight sport codes that comprised men and women participants. Birth quartile graphs for netball (women players only) and for rugby and cricket (men only) complete the figure.

The birthdate distribution of the HP-student-athletes differed from the student cohort. There were no RAEs in 2016 and 2018, despite consistent Q1 and Q2 over-representation. RAEs were more prevalent among the men compared to the women student-athletes. Between-sex differences (medium effect) were noticeable in 2016 only. A ‘spike’ was noted during Q2 in 2017, before normalising again in 2018. From 2019 onwards, the relative distribution in Q1 and Q2 HP-student-athletes increased for both sexes. Among the women student-athletes, a substantial increase was evident in those born during Q2 over the six years. There were no between-sex differences when men and women student-athletes from the same sport code were compared.

## Discussion

To the best of our knowledge, this is the first study to investigate the prevalence and magnitude of the RAE among South African university student-athletes. The RAE was more pronounced among the student-athlete sample compared to the age-matched student cohort. Interestingly, the birth distribution of the student population was slightly skewed towards relatively older students. The prevalence of the RAE among the student population, albeit small, would extend to the HP-student-athlete sample, increasing the likelihood that more Q1 and Q2-born athletes would be competing at this level.

The RAE was only occasionally observed among the HP student-athletes, but the prevalence was inconsistent across the respective years and sports codes. It was more commonly not prevalent than prevalent when the respective subgroups were compared. The HP student-athletes’ birthdate distribution differed significantly from that of the general student cohort, respectively for two (women sample) and three (men sample) of the six years under investigation. The RAE was only prevalent in three of the sport codes: swimming, cricket, and rugby, and there were no sex differences among the HP student-athletes. The actions of different social agents and contextual factors (e.g. developmental pathway, the level of competitiveness and sports popularity) may have contributed to these sport-specific findings.^[4,12]^ The initial selection bias may have merely perpetuated over time.^[5]^ By doing better, relatively older athletes probably received more rewards for their accomplishments, leading to greater psychosocial investment and a better prospect of retaining their participation status, resulting in the RAEs still being prevalent at university sports levels.^[4,6]^

The findings for swimming (i.e. more Q2 than Q1-born swimmers) are atypical and may be attributed to a higher likelihood of an unusual distribution by mere chance, due to the small sample size. Still, there was a bias towards swimmers born in the first half of the year.

Structural changes to the South African first-class cricket competition (i.e. cutting the 11 professional teams to six franchises, thereby reducing the viable development pathways) may have contributed to the RAE observed among the cricket players.^[13]^ Once relatively younger participants deviate from the traditional player pathway, they might find themselves in a development and learning environment against weaker competition and restricted opportunities for progression.^[13,14]^

The RAE observed in this study resonates with previous high school^[15]^ and senior^[6]^ rugby union research in South Africa, thereby adding more information about the pathway to professional rugby. Rugby union is a strong candidate for RAE prevalence based on high physicality (task constraint), cultural relevance and popularity (environmental constraints).^[2,6]^ A residual bias may accumulate from being selected early in the process. Subsequently, fewer relatively younger players may come through the tertiary education pathway.^[13]^

It is difficult to explain the absence of the RAE in soccer and basketball, considering the consistent prevalence reported in these sports in other contexts.^[12,16]^ If students only take up sport later in life, i.e. post-puberty, there could be fewer development variations (e.g. weight, height) when they reach the university sports level. This may reduce physical selection biases and the prevalence of the RAE. Additionally, in comparison to rugby union and cricket, few soccer and basketball players use university sports as a springboard to elite sports. Sports like basketball and soccer may adopt a more flexible approach, where university coaches tend to accept almost any student who wants to be part of the programme, which encourages more students to join these programmes regardless of their initial skill and/or experience, thereby possibly moderating the RAE.

The data showed that only 2% of the student population was part of the HP-student-athlete programme. Women were under-represented in the HP-student-athlete cohort (32% women vs 68% men). This is concerning, considering there were more women than men students (approximately 57% vs 43%, respectively), and raises questions about the under-representation of women in university sports. The differential distributions observed in women HP-student-athletes could be explained by socialisation or a self-restriction process. The “gender inappropriate” stigma attached to the female sport may have weakened results, allowing the relatively older woman and relatively younger student-athletes to continue their participation.

Psychological perspectives embrace the notion of the self-fulfilling prophecy, i.e. the greater the expectation (self-expectations, coach or parent expectations) placed on the player, the greater the achievement result.^[5]^ Studies revealed that coaches held greater expectations of participants born in the first quarter (Q1) of the year than those born in the fourth quarter (Q4).^[17]^ The support provided to athletes during key developmental periods and the developmental experiences created during practice sessions and matches influence their transition and progression.^[1,18]^ Hence, to limit the possible negative consequences of the RAE, swimming, rugby, and cricket administrators should offer diverse solutions to benefit all participants during different participation and development phases.

Talent selection programmes should incorporate a broad range of selection criteria including objective assessments of physical attributes, technical skills, and psychosocial characteristics. Considering that relatively younger players can still reach top-level senior sports, practitioners should consider the delayed development trajectories of some of the young participants and support participants as they transition from high school to university teams. This support is needed both before and once they arrive at university.

Although several solutions have been proposed for youth sports,^[19]^ few have been implemented successfully or tested empirically. Whilst raising awareness is important to address the RAE, it is likely to be insufficient. Moreover, it would be naive to enforce any of the earlier practical recommendations as solutions to reduce this phenomenon because of the absence of direct evidence that their application will reduce the effect. Furthermore, the current findings were limited to information on date of birth, sports codes and sex. It may be too late to impel such interventions at the university sport participation level. Focusing on developing a broader understanding of the processes influencing early and late developing student-athletes may be more appropriate.

## Conclusion

A small RAE was observed among the general student cohort. Analyses of the subgroups revealed inconsistent annual variations among the HP-student-athletes. The RAE was further confined to swimming, cricket, and rugby only, and there were no sex differences in the HP-student-athlete cohort. The observed RAE exemplifies a social inequality that inhibits the prospect of immediate and long-term participation in university HP sport. Even though South African student-athletes are seldom professionals, equal opportunities should be given to everyone to become an HP student-athlete, regardless of date of birth. Even if this bias is unintended, it should be prudently assessed, given the rewarding nature of some sport codes (e.g. access to high-quality resources, television coverage, recognition, financial and academic support). The prevalence of the RAE in these sports may point toward underlying mechanisms and problems with the talent identification, selection, and youth sport development initiatives.

A limitation to this study is the small sample size (especially when split into sport codes). Whilst the present study is representative of student-athletes from a South African university and provides information on the general prevalence of the RAE at this competitive level, it is not comprehensive. Findings from this study are therefore context-specific and should not be generalised to studies from other universities or countries.

Future studies should examine the mechanisms responsible for the prevalence of RAE or the lack thereof at various participation levels (e.g. primary school, high school, and sports academies). Though not examined in this study, it is reasonable to assume a degree of interaction among various constraints. Various individual physical abilities and psychological skills, tasks (playing position, participation level and physicality of the sport), and environmental constraints (popularity of the sport, coach and family influence, sport-code rules, and policies) should be considered and measured explicitly to gain a better understanding of their association with the RAE. Our understanding of these interactions remains limited. Studies may also benefit from triangulating findings from qualitative and quantitative sources and should utilise a sound theoretical framework, such as the Athletic Talent Development Environment model.^[20]^

## Figures and Tables

**Table 1:** Results from the chi-square test (χ^2^) of independence for between-group differences for each sex and academic year

Academic Year	General-student-cohort N (% in group)	HP-student-athletes N (% in group)	χ2 (df = 3)	p-value	Cramer’s V
Men 2016	8 915 (44)	163 (69)	5.59	0.13	0.02
Men 2017	2 413 (44)	156 (80)	9.87	0.05*	0.05
Men 2018	2 262 (43)	85 (67)	3.96	0.27	0.02
Men 2019	2 202 (43)	50 (49)	9.64	0.02*	0.05
Men 2020	2 336 (43)	108 (68)	6.18	0.10	0.04
Men 2021	2 336 (43)	82 (64)	10.17	0.02*	0.05

Women 2016	11 315 (56)	74 (31)	11.42	< 0.01*	0.03
Women 2017	3 107 (56)	40 (20)	0.01	0.99	0.00
Women 2018	3 045 (57)	42 (33)	2.49	0.48	0.00
Women 2019	2 929 (57)	53 (51)	3.85	0.28	0.02
Women 2020	3 080 (57)	50 (32)	8.37	0.04*	0.04
Women 2021	3 115 (57)	47 (36)	4.39	0.22	0.02

* indicates significant differences (p < 0.05). % represent the percentage of total men and women participants per group in an academic year.

**Table 2:** Results from the Fisher’s exact test and residuals for each sport code

Sport code	Men (n)	Women (n)	Total (N)	p-value	Q1 Residual	Q2 Residual	Q3 Residual	Q4 Residual
Athletics	43	47	90	0.58	−1.13	−0.37	−0.70	0.12
Basketball	34	33	67	0.77	0.20	0.01	0.59	−0.84
Football	78	17	95	0.36	1.43	−0.21	−0.31	−1.00
Hockey	65	68	133	0.18	1.16	0.92	−0.86	−1.33
Swimming	34	38	72	<0.01*	0.33	1.82	0.28	−2.57#
Tennis	20	20	40	0.79	0.35	0.59	−0.61	−0.37
Cycling	23	10	33	0.12	−0.65	−0.12	2.04#	−1.29
Water Polo	11	5	16	0.81	0.80	−0.52	0.02	−0.34
Netball	0	67	67	0.54	0.66	0.74	−0.64	−0.84
Rugby	259	1	260	<0.01*	2.00#	1.66	−1.27	−2.61#
Cricket	77	0	77	<0.01*	2.87#	1.47	−2.08#	−2.52#

* indicates significant differences (p < 0.05);

# indicates significant residuals (± 1.96).
